# General practitioners’ hypertension knowledge and training needs: a survey in Xuhui district, Shanghai

**DOI:** 10.1186/1471-2296-14-16

**Published:** 2013-01-28

**Authors:** Qian Chen, Xiangjie Zhang, Jie Gu, Tianhao Wang, Yuan Zhang, Shanzhu Zhu

**Affiliations:** 1 General Practice Department, Zhongshan Hospital, Fudan University, 180 Fenglin Road, Xuhui District, Shanghai, 200032, China

**Keywords:** Awareness, General practitioner, Hypertension, Prevalence, Treatment

## Abstract

**Background:**

Hypertension prevalence is high in China, while patients’ levels of hypertension awareness, treatment and control are low. General practitioners’ knowledge and training relating to hypertension prevention may be an important related factor. We aimed to investigate general practitioners’ knowledge of hypertension prevention and potential training needs.

**Methods:**

A questionnaire survey was conducted among all general practitioners at five community health service centers selected by convenience sampling. A total of 160 questionnaires were distributed and 147 were returned (response rate 91.9%) The questionnaire included general information; 12 subjective questions on health promotion, education and training needs; and 19 objective questions in 5 domains (epidemiology, diagnosis, treatment, referral and community management) measuring knowledge of hypertension prevention and treatment.

**Results:**

The major difficulties in health education practice for general practitioners were poor patient compliance (77.6%) and lack of medical consultation time (49.0%). The average accuracy rate of hypertension prevention knowledge was 49.2%, ranging from 10.5% to 94.7%. The factors associated with accuracy rate were physician’s education level (medical university vs. professional school, β = 13.3, *P* = 0.003), and type of center (training base vs. community healthcare center, β = 12.3, *P* < 0.0001). Most physicians (87.8%) reported being willing to attend training courses regularly and the preferred frequency was once every 2 ~ 3 months (53.5%). The preferred course was medical treatment of hypertension (82.3%) and the most favored training approach was expert lectures (80.3%).

**Conclusions:**

The knowledge level of hypertension prevention is low among general practitioners in urban settings. Physicians working in community clinics where they participate in a series of teaching, assessing and evaluating systems for hypertension prevention perform better than those in general healthcare centers who lack specific training. Continuing hypertension education is urgently needed to ensure that physicians in general practice are aware of and adhere to the national hypertension prevention guidelines.

## Background

Hypertension is a common chronic disease worldwide and a major risk factor for cardiovascular disease. National survey data suggest that the prevalence of hypertension in the Chinese adult population has quadrupled from 5% in 1959 to nearly 19% in 2002 [1]. In 2002, 153 million Chinese adults were hypertensive, with overall prevalence 24%, including higher prevalence in urban than in rural areas in men (23% vs. 18%) and women (18% vs. 16%) [2]. A report of the China Multi-Center Study of Cardiovascular Epidemiology showed that 24% of 1000 participants from 13 different study populations between 1992 and 1998 had hypertension, with prevalence 25.4% higher in urban than in rural areas; patients’ awareness rate (aware of having a history of high blood pressure) was 42.6%, treatment rate (currently using antihypertensive drugs) 31.1% and control rate (blood pressure controlled under 140/90 mmHg) 6.0% [3]. A cross-sectional study in April 2007 in rural Shandong Province, China, found hypertension prevalence to be high (43.8%), while awareness (26.2%), treatment (22.2%) and control of hypertension (3.9%) were unacceptably low [4]. In Guangdong province, a province in southern China, the prevalence of hypertension in this population was 20.5%, which translates to a total of 9.8 million adults who suffered from hypertension [5]. In urban regions, the prevalence of awareness, treatment, and control of hypertension among the hypertensive patients were 42.8, 37.9, and 13.5%, respectively, which were higher than that among patients living in rural areas [5]. The reports above clearly indicate that prevalence in China is increasing—and that public health measures are needed to enhance awareness, treatment and control of hypertension.

Low awareness of hypertension prevention among Chinese physicians may be an important factor relating to increasing prevalence of hypertension. In China, general practitioners provide comprehensive healthcare for people of all ages with all diseases. They function in the same way as family physicians (FPs), primary care physicians or general practitioners (GPs) in other countries. However, primary care is provided by general practitioners with different levels of education; they can either be graduates of a medical college with no postgraduate training, or they can be doctors with postgraduate training and specialization [6]. Although patients seek help from general practitioners for acute illness, patients report doubting the quality of general practitioners’ care for chronic illness and have more confidence in hospital clinics, which are perceived to have more qualified physicians and more modern equipment and procedures [6]. Therefore, many patients do not seek regular care from general practitioners, which can mean delayed diagnosis for asymptomatic conditions that may include hypertension.

The first edition of the “Chinese Hypertension Prevention Guide” was officially released in December 2009 [7]. It was developed jointly by the Disease Control and Prevention Bureau of the Ministry of Health, National Center for Cardiovascular Disease and the Chinese Hypertension Alliance as China’s first authoritative practical hypertension prevention and treatment guide. It establishes adults’ target blood pressure as SBP/DPB < 140/90 mmHg in uncomplicated hypertension; < 150/90 mmHg for adults > 65 years; and < 130/80 mmHg for those with diabetes, coronary heart disease or renal disease. It also advises on antihypertensive drug use and emphasizes lifestyle modification such as sodium restriction, smling cessation, weight loss, reduced alcohol consumption and increased dietary potassium and physical activity as prevention and control measures. As such, the Chinese guide compares favorably to other national and international hypertension guidelines followed by general practitioners in other countries. Flynn and Flavin (2012) report that clinical guidelines for hypertension and ambulatory blood pressure in Ireland emphasize ambulatory blood pressure monitoring to improve blood pressure control in patients receiving primary care, but guideline use is limited by physician’s lack of time and costs of monitoring [8]. Guidelines are widely applied in Sweden but general practitioners report accepting higher blood pressure levels than clinical guidelines advise are appropriate [9]. In the United States, guidelines are provided through continuing medical education and are shown to influence provider practice patterns and patient outcomes, serving as a cost-effective strategy for prevention [10]. The 2009 Canadian Hypertension Education Program published its comprehensive recommendations for hypertension prevention and management, which included specific lifestyle modifications to restrict dietary sodium, perform aerobic exercises, maintain healthy body weight and waist circumference; detailed dietary recommendations, alcohol limitations and stress management techniques were also included as well as recommended pharmacologic agents [11]. The National Clinical Guideline Centre in the UK updated its hypertension guidelines in 2011; it comprises evidence-based advice on the care and treatment of adults with primary hypertension, including new, updated diagnosis, antihypertensive drug treatment and monitoring [12]. Most guidelines are updated annually.

The Chinese Hypertension Prevention Guide is directed to the general population (urban communities and rural health service organizations) and is designed to serve as the standard teaching guide for training primary care physicians. However, goals to increase general practitioners’ awareness of the Guide, to carry out hypertension education, prevention and control in the communities and to improve hypertension awareness, treatment and control rates in hypertensive patients remain difficult tasks to accomplish nationwide. As a preliminary approach, this study aimed to investigate general practitioners’ knowledge of hypertension, understanding and use of the national hypertension prevention and control guidelines, and potential training needs. Results may provide a basis for developing a hypertension training program targeted at community physicians.

## Methods

### Subjects

Five community health service centers in Xuhui District, Shanghai, were selected by convenience sampling based on their accessible urban setting; geographic location of the centers were considered and included one suburban area, two urban areas (not downtown) and two downtown areas to represent the general metropolitan area, which has a similar health network to other regions of China. All general practitioners in the centers were invited to participate in this study. A questionnaire survey was conducted among general practitioners at those centers from March 2011 to June 2011. A total of 160 questionnaires were distributed and 147 were returned (response rate 91.9%).

### Definitions

#### General practitioner

General practitioners in China are similar to family physicians (FPs) or general practitioners (GPs) in other countries and provide comprehensive healthcare for people of all ages, sexes, diseases and parts of the body.

#### Physicians’ titles and medical education levels

In China, physicians include junior, attending, and senior physicians, corresponding to resident, attending physician and chief physician or deputy chief physician in other countries. Physicians’ medical education levels vary from: (a) graduates of a 5-year regular medical university (“bachelors”) equal to having a Bachelor of Medicine degree without clinical internship or research work; (b) graduate students with Master of Medicine or Doctor of Medicine degrees who study and perform clinical and research work for 3 to 6 years; and (c) graduates of professional school (equal to senior high school level) and medical college (3-year training in medicine after high school), a category that is being reduced.

#### Healthcare network and work stations

The network includes small community clinics set up as work stations by community health service centers providing public health services and patient education in residential communities. The clinics’ main function is to facilitate management and follow-up of residents’ chronic diseases (e.g., hypertension, diabetes mellitus), including measuring blood pressure, monitoring blood glucose, dispensing medicine and performing injections or intravenous infusion. Each site has 4 to 5 health service personnel (e.g., attending physician, 1 to 2 residents and 1 to 2 nurses) as well as out-patient clinics and wards. Community health service centers also conduct community workshop to provide academic exchange between community general practitioners and health education for community residents and patients with chronic diseases.

#### Scope of medical services

The five community health centers included in this study had similar functional characteristics but affiliated medical facilities in the suburbs and urban/downtown areas had differences in the scope of clinical diagnosis and treatment, as follows: (1) *Full medical service*: Suburban community healthcare centers have fewer surrounding secondary and tertiary hospitals/medical center to share medical services and therefore provide full medical service with a greater workload, including more patients and more types of diagnosis and treatment. (2) *Basic healthcare*: Downtown community healthcare centers provide basic medical services with a smaller workload, including fewer patients, fewer types of diagnosis and treatment. Urban community healthcare centers provide medical services between basic and full service.

### Instrument

A questionnaire survey was conducted to investigate the hypertension knowledge and training needs of community general practitioners. The questionnaire comprised three sections (refer to Appendix A for full questionnaire): (A) General information, which included physicians’ baseline characteristics; health promotion, education and training needs; and knowledge of hypertension prevention and treatment; (B) Health Education and Training Needs, which included hypertension promotion, education of community general practitioners and physicians’ hypertension-related training needs. It originally included 12 subjective questions presented as true/false and multiple choice questions but two items were deleted due to the low in CVI; and (C) Knowledge of hypertension prevention and control, which included 5 domains with 19 items derived from the China Hypertension Prevention Guide [7]. Domains were epidemiology with 1 item, diagnosis with 6 items, treatment with 9 items, two-way referral with 2 items and community management with 1 item. Content validity of the questionnaire was calculated as previously described by Rubio et al. [13]. Content validity was evaluated by six experts, including one professor and five senior physicians with expertise in general practice. Content validity indices (CVI) of Section B and Section C were 0.967 and 0.974, respectively.

### Data collection

The questionnaires were distributed and interpreted by survey administrators. Participants were asked to complete the self-reported questionnaire without discussing with others. The completed questionnaires were collected personally by survey administrators along with signed informed consent to participate. The survey administratos were faculty of community health service centers who had received a short training course about the study purpose, questionnaire content and how to answer the questionnaire. Researchers standardized the procedure to distribute and recover the questionnaire. The authenticity and validity of the returned questionnaires were verified by researchers in research team (i.e., all questions answered by the respondent) before performing data processing and statistical analyses.

### Statistical analysis

Continuous variables were summarized by mean ± SD and categorical variables were expressed by frequencies and percentages. Differences in the distribution of physicians’ demographic characteristics among the five participating community healthcare centers were detected by Chi-square test or Fisher’s exact test, as appropriate. The accuracy rate of hypertension-related knowledge was calculated as number of correctly answered items divided by total number of items for each physician. For example, if a physician correctly answered 12 items among a total of 19 items, his accuracy rate of hypertension-related knowledge would be 12/19 = 63.2%. To investigate factors associated with the accuracy rate of hypertension-related knowledge, the generalized estimating equation (GEE) model was applied to accommodate the correlated data of physicians clustered within the same healthcare center. The accuracy rate served as a continuous dependent variable, and demographic characteristics of physicians as well as characteristics of community healthcare centers served as independent variables. Univariate and multivariate GEE models with identity links were used to calculate unadjusted and adjusted mean differences and standard errors (SE) of the accuracy rate, compared to the appropriate reference group. The multivariate GEE model was constructed using backward selection procedure, wherein variables that did not improve model fit at *P* < 0.05 were discarded, but age of physicians was always forced into the model for adjustment. All statistical analyses were performed with SAS software version 9.2 (SAS Institute Inc., Cary, NC). A two-tailed *P* < 0.05 indicated statistical significance.

## Results

### Baseline characteristics of general practitioners

The baseline characteristics of general practitioners are summarized in Table 1. A total of 147 physicians completed questionnaires. Mean age of subjects was 39.8 ± 10.9 years (range: 25–62 years). More female physicians (60.7%) were included than males (39.3%). Less than half of physicians (49.3%) were attending physicians, followed by junior physicians (34.7%) and senior physicians (16.0%). Most physicians (41.3%) worked at community health stations. In terms of education level, a majority of physicians (78.2%) graduated from medical university. Among the 5 community healthcare centers, significant differences were found in distributions of age (*P* = 0.018), title (*P* = 0.003), working position (*P* < 0.0001) and number of years worked (*P* = 0.001).

**Table 1:** Baseline characteristics of general practitioners in different community healthcare centers

**Characteristics**		**Community healthcare centers**					
	**Total**	**1**	**2**	**3**	**4**	**5**	**P-value**
	**(n = 147)**	**(n = 40)**	**(n = 38)**	**(n = 12)**	**(n = 32)**	**(n = 25)**	
Age ^1^(year), n(%)							0.018^†^
21 ~ 30	36 (25.5)	6 (16.2)	6 (17.1)	1 (8.3)	10 (31.3)	13 (52.0)	
31 ~ 40	53 (37.6)	9 (24.3)	17 (48.6)	7 (58.3)	12 (37.5)	8 (32.0)	
41 ~ 50	17 (12.1)	7 (18.9)	5 (14.3)	1 (8.3)	2 (6.3)	2 (8.0)	
≥ 51	35 (24.8)	15 (40.5)	7 (20.0)	3 (25.0)	8 (25.0)	2 (8.0)	
Gender ^2^, n(%)							0.121^¶^
Male	57 (39.3)	12 (30.0)	16 (44.4)	3 (25.0)	18 (56.3)	8 (32.0)	
Female	88 (60.7)	28 (70.0)	20 (55.6)	9 (75.0)	14 (43.8)	17 (68.0)	
Title ^3^, n(%)							0.003^¶^
Junior physician	50 (34.7)	7 (18.0)	9 (25.0)	3 (25.0)	14 (43.8)	17 (68.0)	
Attending physician	71 (49.3)	22 (56.4)	20 (55.6)	6 (50.0)	16 (50.0)	7 (28.0)	
Senior physician	23 (16.0)	10 (25.6)	7 (19.4)	3 (25.0)	2 (6.3)	1 (4.0)	
Working position ^4^, n(%)							< .0001^¶^
Out-patient	47 (32.9)	12 (31.6)	15 (41.7)	1 (8.3)	19 (59.4)	0 (0.0)	
Ward	37 (25.9)	14 (36.8)	9 (25.0)	7 (58.3)	7 (21.9)	0 (0.0)	
Community health station	59 (41.3)	12 (31.6)	12 (33.3)	4 (33.3)	6 (18.8)	25 (100.0)	
Education level ^5^, n(%)							0.066^†^
Professional school	9 (6.3)	5 (13.2)	1 (2.8)	0 (0.0)	2 (6.3)	1 (4.0)	
Medical College	17 (12.0)	8 (21.1)	3 (8.3)	0 (0.0)	6 (18.8)	0 (0.0)	
Medical University	111 (78.2)	23 (60.5)	31 (86.1)	10 (90.9)	24 (75.0)	23 (92.0)	
Graduate school	5 (3.5)	2 (5.3)	1 (2.8)	1 (9.1)	0 (0.0)	1 (4.0)	
Years working ^6^, n(%)s							0.001^†^
≤ 10	48 (33.6)	5 (12.8)	9 (25.0)	3 (25.0)	14 (45.2)	17 (68.0)	
10–19	48 (33.6)	11 (28.2)	17 (47.2)	5 (41.7)	10 (32.3)	5 (20.0)	
20–29	15 (10.5)	8 (20.5)	3 (8.3)	2 (16.7)	1 (3.2)	1 (4.0)	
≥ 30	32 (22.4)	15 (38.5)	7 (19.4)	2 (16.7)	6 (19.4)	2 (8.0)	

^†^Fisher’s exact test; ^¶^ Chi-square test.

^1^ Two subjects with missing values. ^2^ Six subjects with missing values. ^3^ Three subjects with missing values. ^4^ Four subjects with missing values. ^5^ Five subjects with missing values. ^6^ Four subjects with missing values.

### Health education for hypertension practice among general physicians

Almost all physicians (99.3%) believed that changing unhealthy lifestyles will help control blood pressure, and all general practitioners (100%) intended to practice health education for hypertensive patients during their clinic work (Table 2). Physicians encountered 4 main difficulties in health education practice, including poor patient compliance (77.6%), lack of medical consultation time (49.0%), lack of related educational knowledge (25.9%), and lack of technique of behavioral medicine (e.g. communication behaviors between clinician and patient within the field) (18.4%). Most physicians (81.0%) believed that health education would change lifestyle of hypertensive patients to some extent, and approximately 7 in 10 physicians (71.4%) reported spending one third to one quarter (1/3 ~ 1/4) of their practice time in medication consultation with hypertensive patients. Questions from the questionnaire are shown in Table 2; the entire questionnaire can be viewed in Appendix A.

**Table 2:** Health education for hypertension and demand for hypertension training by general practitioners in community healthcare centers

**Questions and responses**	**n (%)**
***Health Education for Hypertension***	
1. Do you think that changing unhealthy lifestyle helps control blood pressure?	
Yes	146 (99.3)
No	0 (0.0)
Missing	1 (0.7)
2. Do you intend to practice health education for patients with hypertension during your regular clinic work?	
Yes	147 (100.0)
No	0 (0.0)
3. What is the difficulty in practicing health education to patients with hypertension? (multi-choice)	
Lack of related educational knowledge	38 (25.9)
Poor compliance of patients	114 (77.6)
Lack of technique of behavioral medicine	27 (18.4)
Lack of medical consultation time	72 (49.0)
Other	3 (2.0)
4. Do you think that health education will change the lifestyle of patients with hypertension?	
Yes, determinatively	27 (18.4)
Yes, to some extent	119 (81.0)
No	0 (0.0)
Missing	1 (0.7)
5. How much time did you spend on practicing health education to patients with hypertension in terms of time for medical consultation?	
3/4 or more	5 (3.4)
1/2	31 (21.1)
1/3	46 (31.3)
1/4	59 (40.1)
Less or none	6 (4.1)
***Demands for hypertension education and training***	
6. Did you ever attend any training course on hypertension prevention?	
Yes	136 (92.5)
No	6 (4.1)
Missing	5 (3.4)
7. Would you like to attend training courses on hypertension prevention regularly?	
Yes	129 (87.8)
No	13 (8.8)
Missing	5 (3.4)
8. What is the most appropriate frequency of related training course? †	
Weekly	2 (1.6)
Bi-weekly	9 (7.0)
Monthly	47 (36.4)
Every 2 ~ 3 months	69 (53.5)
Don’t need training course	2 (1.6)
9. What topics should be covered in the related training course? (multi-choice)	
Diagnosis and evaluation of hypertension	82 (55.8)
Medical treatment of hypertension	121 (82.3)
Education for hypertensive patients (including non-medication treatment)	97 (66.0)
Management of hypertensive patients in community	84 (57.1)
Two-way referral of hypertensive patients	55 (37.4)
Treatment for patients with complication/comorbidity	80 (54.4)
Other	6 (4.1)
10. What kind of format(s) should be used in the related training course? (multi-choice)	
Expert lectures	118 (80.3)
On-site guidance	45 (30.6)
Case study	91 (61.9)
Community workshop	68 (46.3)
Others	1 (0.7)

† Among those want to attend training course regularly (n = 129).

### General practitioners’ demand for hypertension prevention training

A majority of physicians (92.5%) had previously attended training courses on knowledge of hypertension prevention, and most physicians (87.8%) reported being willing to attend training courses regularly (Table 2). Among physicians willing to attend regular training (n = 129), the preferred frequency of training courses was once every 2 ~ 3 months (53.5%), followed by once every month (36.4%). Regarding topics covered in the training course, most physicians would like to learn about medical treatment of hypertension (82.3%), followed by educating the hypertensive patients (66.0%) and management of hypertensive patients in the community (57.1%). Expert lectures (80.3%) and case study (61.9%) were the two most favored training approaches.

### Hypertension prevention knowledge among general physicians

Hypertension prevention knowledge included 5 domains with 19 items derived from the Chinese Hypertension Prevention Guide [7]. Domains were epidemiology with 1 item, diagnosis with 6 items, treatment with 9 items, two-way referral with 2 items and community management with 1 item. Accuracy rates for the five domains were 44.2%, 46.3%, 54.4%, 50.7%, and 21.8%, respectively (Table 3). Among 147 physicians, the average accuracy rate of all 19 items was 49.2%, ranging from 10.5% (correctly answered 2 of 19 items, 2 physicians) to 94.7% (correctly answered 18 of 19 items, 5 physicians). The responses of general physicians to each item in this category are shown in Appendix B (Additional file 1: Table S1).

**Table 3:** Accuracy rate in different domains of questionnaire for knowledge of hypertension

**Domain of items**	**No. of items**	**No. of subjects**	**Accuracy rate† (%)**
Epidemiology	1	147	44.2
Diagnosis	6	147	46.3
Treatment	9	147	54.4
Two-way referral	2	147	50.7
Community Management	1	147	21.8
Total	19	147	49.2

† Average of 147 physicians.

When general physicians were asked the question about current prevalence of hypertension in China, only 44.2% (65/147) answered correctly. Regarding diagnosis, 44.2% (65/147) of general physicians mistakenly chose the item “For patients without the use of any antihypertensive drugs, hypertension can be diagnosed if two measurements on different days show systolic blood pressure > 140 mmHg and/or diastolic blood pressure > 90 mmHg.” For blood pressure measuring procedure, 45.6% (67/147) general physicians mistakenly chose the item “The time interval between two blood pressure measurements is 1 to 2 minutes.” Only 49.7% (73/147) of general physicians correctly chose the item “The blood pressure of elderly patients (age ≥ 65 years) with hypertension should be reduced to below 150/90 mmHg.” For drug-related questions, 27.9% (41/147) of general physicians mistakenly thought the indications of dihydropyridine calcium antagonists was left ventricular dysfunction, and 23.1% (34/147) mistakenly thought the taboo against the use of diuretics was diabetic nephropathy. For management grade of hypertensive patients, 36.7% (54/147) of general physicians mistakenly chose the item “For patients with target organ damage who are managed by grade, the management grade can be adjusted based on the actual situation after 1 year of management.” For ambulatory blood pressure monitoring, 23.1% (34/147) of general physicians mistakenly thought that normal ambulatory blood pressure values in China are: 24-h average < 130/80 mmHg, daytime average < 140/90 mmHg, night time average < 120/70 mmHg.

### Factors associated with accuracy rate of hypertension prevention knowledge

The factors associated with accuracy rate of hypertension prevention knowledge were investigated using univariate and multivariate GEE models (Table 4). In univariate analysis, physicians with younger age, graduated from medical university, fewer years working experience and those from training base were significantly associated with higher accuracy rate. After backward selection procedure, only physician’s age, education level, and type of center were included in the final multivariate model. After controlling for other factors in the multivariate model, physician’s age was no longer associated with accuracy rate, but education level (medical university vs. professional school, adjusted mean difference: β = 13.3, *P* = 0.003) and type of training center (training base vs. community healthcare center, adjusted mean difference: β = 12.3, *P* < 0.0001) were still significant factors associated with accuracy rate.

**Table 4:** Univariate and multivariate factors associated with accuracy rate in knowledge of hypertension using GEE models

	**n**	**Accuracy rate (%)**	**Univariate**	**P-value**	**Multivariate**^**†**^	**P-value**
		**mean ± SD**	**β**^**‡**^**(SE)**		**β**^**¶**^**(SE)**	
*Characteristics of physician*						
Age ^1^(year)						
21 ~ 30	36	55.1 ± 17.5	reference	--	reference	--
31 ~ 40	53	54.1 ± 21.3	−1.0 (4.7)	0.832	1.7 (4.1)	0.686
41 ~ 50	17	41.8 ± 23.1	−13.3 (5.4)	0.013	−7.9 (7.1)	0.263
≥ 51	35	42.0 ± 24.1	−13.2 (6.3)	0.036	1.0 (5.6)	0.859
Gender ^2^						
Male	57	46.9 ± 20.6	−4.2 (5.3)	0.424		
Female	88	51.1 ± 23.1	reference	--		
Title ^3^						
Junior physician	50	51.6 ± 19.4	reference	--		
Attending physician	71	49.0 ± 23.4	−2.6 (4.5)	0.562		
Senior physician	23	47.6 ± 24.0	−4.0 (10.8)	0.712		
Working position ^4^						
Out-patient	47	46.6 ± 25.4	reference	--		
Ward	37	49.8 ± 20.8	3.2 (13.8)	0.817		
Community health site	59	52.5 ± 19.9	6.0 (8.3)	0.472		
Education level ^5^						
Professional school	9	40.4 ± 18.2	reference	--	reference	--
Medical college	17	35.0 ± 25.7	−5.4 (8.8)	0.543	−2.4 (8.1)	0.769
Medical university	111	53.4 ± 20.9	13.0 (6.1)	0.033	13.3 (4.5)	0.003
Graduate school	5	41.1 ± 17.6	0.7 (9.5)	0.941	4.5 (11.1)	0.684
Years working^6^						
< 10	48	54.7 ± 19.0	reference	--		
10 ≦ ~ <20	48	53.6 ± 20.6	−1.1 (2.7)	0.684		
20 ≦ ~ <30	15	45.6 ± 20.6	−9.1 (9.7)	0.349		
≧ 30	32	38.8 ± 25.7	−15.9 (4.4)	0.0003		
*Characteristics of primary care center*						
Type of center						
Community Healthcare center	72	45.1 ± 19.9	reference	--	reference	--
Community Teaching base	50	48.3 ± 25.1	3.2 (4.4)	0.469	−1.0 (3.7)	0.796
Training base	25	62.7 ± 16.9	17.6 (3.0)	<0.0001	12.3 (1.7)	< 0.0001
Location						
Suburban	32	49.8 ± 12.5	reference	--		
Urban (not include downtown)	50	48.3 ± 25.1	−1.5 (3.3)	0.645		
Downtown area	65	49.6 ± 23.7	−0.3 (7.2)	0.969		
Scope of medical seervice						
Basic healthcare	65	49.6 ± 23.7	reference	--		
Between basic and full service	50	48.3 ± 25.1	−1.2 (7.9)	0.875		
Full medical service	32	49.8 ± 12.5	0.3 (7.2)	0.969		

^†^ In the multivariate model, only data of 139 physicians were analyzed.

^‡^ Unadjusted mean difference compared to referent group.

^¶^ Adjusted mean difference compared to referent group.

^1^ Two subjects with missing values. ^2^ Six subjects with missing values. ^3^ Three subjects with missing values. ^4^ Four subjects with missing values. ^5^ Five subjects with missing values. ^6^ Four subjects with missing values.

## Discussion

This study investigated general practitioners’ needs for continuing education and knowledge of hypertension prevention, which are closely associated with their understanding and use of the national hypertension prevention and control guidelines. For the questions regarding hypertension prevention knowledge, the average accuracy rate of all 19 items was 49.2%, ranging from 10.5% to 94.7%, suggesting that the knowledge level of general practitioners in Xuhui District, Shanghai was low. Physicians with better understanding of hypertension (i.e., higher accuracy in questionnaire answers) were more likely to be younger, with at least an undergraduate education level (i.e., having a bachelor or higher in medical degree vs. professional school education), fewer years of working experience (indicating more recent education and training) and working in a training-base. Regarding training needs, most general practitioners reported being willing to attend training courses regularly. Their preferred frequency of training courses was once every 2 ~ 3 months, and the most preferred course was “medical treatment of hypertension” followed by “education for hypertensive patients (including non-pharmacologic lifestyle treatment). The most favored training approaches were expert lectures and case study. These findings indicate that physicians are eager to receive more training on addressing hypertension prevention in their communities. The increasing prevalence of hypertension and the related high risk for cardiovascular disease is a growing public health problem. Based on the quantity and structure of the Chinese population in 2010, about 200 million patients are projected to have hypertension in China, or about 2 hypertensive patients in every 10 adults [2]. Causes of the high prevalence of hypertension may be attributed to various factors, including socio-demographic factors such as older age, lower educational levels, lower income and living alone [14]. The public’s lack of awareness of the presence of hypertension and lack of compliance with treatment after diagnosis are also implicated [15]. Genetic causes have been indicated as well, including the α-adducin Gly460Trp gene polymorphism, which is linked to essential hypertension susceptibility, especially in Han Chinese [16]. To address the high prevalence of hypertension, the Chinese government has officially released a “Chinese Hypertension Prevention Guide,” which is the first authoritative practical hypertension prevention and treatment guide available to Chinese practitioners and the public [7]. The guide is directed to urban communities and rural health service organizations to serve as the standard teaching guide for training primary care physicians. However, the knowledge level of general practitioners in Xuhui District, Shanghai as evidenced in the present study, is relatively low, suggesting that a considerable percentage of general practitioners do not carefully read and adhere to the published guidelines.

Barriers to adherence to national guidelines in China were suggested by results of this study. General practitioners reported four main obstacles in their practice: poor patient compliance, not enough time for medical consultation, lack of their own knowledge related to hypertension and lack of technique in behavioral medicine. Although most physicians thought that health education could influence lifestyle changes in hypertensive patients to some extent, 7 in 10 physicians reported spending only one third to one quarter of their practice time in medication consultation to educate patients about lifestyle changes that may help to reduce blood pressure and benefit their health status. Similar barriers to adherence to national and international hypertension guidelines are reported by general practitioners in other countries. The main obstacles to guideline use in Ireland as support for ambulatory blood pressure monitoring is physicians’ time and the costs related to monitoring, as well as a lack of remuneration [8]. In Sweden, general practitioners report accepting higher blood pressure levels than clinical guidelines advise are appropriate [9]. Physicians included in that study reported that the advanced age of older adult hypertensive patients was a barrier to effective pharmacologic management of hypertension and that benefits of pharmacologic treatment are not obvious. About half of the physicians used risk assessment tools and nine out of ten informed their patients about the target levels for blood pressure. In Saudi Arabia, low rates for control of hypertension prompted an evaluation of primary care physicians’ adherence to clinical practice guidelines [17]. In that study, only 5% of physicians measured blood pressure with patients sitting and standing, correct diagnoses of systolic and diastolic blood pressure was inconsistent among physicians, clinical examination items were not completed by all physicians, recommended diagnostic laboratory tests were missed (e.g., serum creatinine, lipid profile) and first-line anti-hypertensive agents (thiazide diuretics) were only prescribed by less than one-fifth of all participating physicians [17]. In Australia, a survey of general practice supervisors and registrars showed that hypertension was the most common problem seen by general practitioners but remained undertreated; reasons for undertreatment included lack of clarity and consistency in evidence-based guidelines, especially regarding first line treatment [18]. However, nearly 75% to 80% of untreated hypertensive adults in an Australian hypertensive medication survey had their blood pressure measured within 12 months preceding the survey [18,19]. In Croatia, the majority of physicians said that they support using guidelines for evaluating cardiovascular risk, but only half of physicians used them and their knowledge of specific guidelines was unsatisfactory [20]. In that study, despite the fact that most risk factors for cardiovascular disease are modifiable and that Joint European guidelines on cardiovascular disease prevention target high-risk patients, the trends in management were described as disappointing, including that guidelines seem to be ignored [20]. Overall, physicians in the above studies seemed doubtful that guidelines actually improve patient outcomes.

In summary, responses from general practitioners in this study implied that they did not conduct hypertension consultations effectively due to lack of consultation time, which was compounded by patients’ poor compliance with physicians’ instructions. Although the healthcare network in China includes community health service centers that provide public health services and patient education in residential communities, with management and follow-up of residents’ chronic diseases (e.g., hypertension, diabetes mellitus) performed by smaller clinics, there is clearly a need for a well-organized program for hypertension monitoring and consultation so that all hypertensive patients can receive necessary attention and education by trained physicians. The need to promote the national guidelines is obvious. The question of how to promote them remains, especially given the structure of the community health centers and the mixed educational backgrounds of general practitioners working in these centers. China has tried to standardize care throughout the national healthcare network and improve care delivered by the community health centers. Our study results do show that physicians working in the training base with a series of teaching, assessing and evaluating systems and settings for hypertension prevention perform better than those that are in general healthcare centers. This makes a strong case for continuing education for general practitioners.

The new, updated Chinese Hypertension Prevention Guide [7] sets the standard for blood pressure in normal adults as 140/90 mm/Hg or below, encourages modification of lifestyle factors by all adults as a preventive measure and stresses the long-term stable control of blood pressure as one of the modifiable risk factors for cardiovascular and cerebrovascular disease, which both have high morbidity and mortality in China. The guidelines could result in improving community health if they could be promoted sufficiently to increase both physician and public awareness. The status of hypertension management in China in 2002 was characterized by patients’ low awareness rate (not aware of their high blood pressure) (44.7%), low treatment rate (poor compliance with prescribed antihypertensive medication) (28.2%) and low control rate (poor achievement of recommended blood pressure control < 140/90 mm Hg) (8.1%) [2]. Since then, a wide range of health education and promotion has been conducted through the efforts of various levels of government and medical organizations, but surveys still show a year-to-year increasing trend in hypertension [1,2], indicating modest success, if any, in improving prevention and control in the Chinese population. The new evidence-based guidelines are available and a strategy for promoting them to physicians must be designed and implemented. Education can possibly be prioritized based on general practitioners’ individual levels of medical education, emphasizing training for hypertension prevention and treatment. Meanwhile, patients’ knowledge and awareness are important to preventing and controlling hypertension and available media should be used to communicate prevention information to the public. A dialogue between patients and general practitioners could ultimately benefit both the health status of the patients and the general practice of community health centers.

The present study is limited because it only surveyed general practitioners in one district of one city in China. Results may not represent the state of general practice throughout the country or in different types of institutions in the national healthcare network. Also, although we evaluated general practitioners’ interest in receiving certain types of education and training to address hypertension in their communities, specific areas of weakness in their knowledge of hypertension were not evaluated in depth. In addition, our survey instrument was self-reported and general practitioners’ hypertension-related skills and knowledge should ideally be measured quantitatively with an instrument designed and validated for that purpose.

## Conclusion

In conclusion, the knowledge level of community general practitioners in Xuhui District, Shanghai, on understanding, preventing and managing hypertension in their communities is limited and their awareness of practice guidelines for hypertension is low. Physicians working in training bases, where they participate in a series of teaching, assessing and evaluating systems for hypertension prevention, had higher accuracy rate in hypettension knowledge than those in general community clinics where they lack specific training. Continuing education is urgently needed to ensure that physicians in general practice are aware of and adhere to hypertension prevention and treatment strategies presented in the Chinese Hypertension Prevention Guide. Increasing general practitioners’ awareness and understanding of hypertension prevention and treatment is necessary to reduce the prevalence of hypertension in China. Further study is needed with a larger sample representing broader geographic distribution. Investigation of existing hypertension awareness programs, monitoring system and their level of success in terms of outcomes and control of hypertension also may be of value.

## Appendix: Appendix A

### Questionnaire: Hypertension Knowledge and training needs for Community General Practitioners

A. General Information

1. Name:

2. Gender: □Male □Female

3. Age: □□

4. Position title: □Junior physician □Attending physician □Senior physician

5. Workplace: □Outpatient clinic □Ward □Community health station

6. Education: □Professional school □Medical college □Medical university □Graduate school

7. Work Experience: □Less than 10 years □10 ~ 20 years □20 ~ 30 years □More than 30 years

B. Health Education and Training Needs

1. Do you think that changing unhealthy lifestyles helps to control blood pressure? □ Yes □ No

2. Do you consciously conduct health education for patients with hypertension in your daily work? □Yes □No

3. What is the difficulty in providing health education during clinical practice to patients with hypertension? (*You can choose more than one answer*.)

(A) Lack of relevant educational knowledge

(B) Poor compliance of patients

(C) Lack of behavioral medicine techniques

(D) Patients’ visits do not allow long enough time

(E) Other

4. Do you think that health education will change the lifestyle of patients with hypertension?

(A) Yes, definitely (B) Yes, to some extent (C) No

5. How much medical consultation time did you spend on providing health education to patients with hypertension?

(A) 3/4 or more (B) 1/2 (C) 1/3 (D) 1/4 (E) Lesser or 0\

6. Have you received any training courses on hypertension prevention and control knowledge? □ Yes □ No

7. Would you like to attend training courses on hypertension prevention regularly?

□ Yes □ No

8. What is the most appropriate frequency of related training course?

(A) Weekly (B) Bi-weekly (C) Monthly (D) Once every 2 ~ 3 months (E) Don’t need training course

9. What topics should be covered in the related training course? (*You can choose more than one answer*.)

(A) Diagnosis and evaluation of hypertension

(B) Medical treatment of hypertension

(C) Education for hypertensive patients (including non-medication treatment)

(D) Management of hypertensive patients in community

(E) Two-way referral of hypertensive patients

(F) Treatment for patients with complications/comorbidity

(G) Other

10. What kind of format(s) should be used in the related training course? (*You can choose more than one answer*.)

(A) Expert lectures (B) On-site guidance (C) Case Study (D) Community workshop (E) Other

C. Knowledge of hypertension prevention and control

1. The epidemiological data show that the current prevalence of hypertension in China is?

(A) 50 million (B) 100 million (C) 160 million (D) 200 million (E) 250 million

2. Which of the following is correct about the diagnosis of hypertension?

(A). For patients without the use of any antihypertensive drugs, hypertension can be diagnosed if two measurements on different days show systolic blood pressure > 140 mmHg and/or diastolic blood pressure > 90 mmHg.

(B). Systolic blood pressure > 140 mmHg and diastolic blood pressure ≤ 90 mmHg is isolated systolic hypertension.

(C). Systolic blood pressure <140 mmHg and diastolic blood pressure > 90 mmHg is isolated diastolic hypertension.

(D). When grading hypertension, if the patient’s systolic and diastolic blood pressure belongs to different grades, a lower grade should be selected.

(E). At the same time as diagnosing hypertension, the risk should be stratified according to patient’s blood pressure levels, existing risk factors, target organ damage, and combined clinical disorders.

3. Which of the following is correct about detecting patients with hypertension in communities? *(You can choose more than one answer.)*

(A.) At all levels of medical institutions, blood pressure should be measured at the first visit of patients over age 35 years.

(B.) For populations susceptible to hypertension (BP 130-139/85-89 mmHg, obesity, etc.), it is recommended that blood pressure should be measured every six months.

(C.) A variety of public places should be used to measure blood pressure, including elderly activity stations, clinics at institutions and blood pressure measuring stations.

(D.) Make plans to measure the blood pressure of all adults in the community, and blood pressure of adults with normal blood pressure should be measured at least once every 2 years.

(E.) Place semi-automatic or automatic electronic blood pressure monitors at a variety of public places to facilitate the public’s self-measurement of blood pressure.

4. For a patient with hypertension whose blood pressure is 165/95 mmHg, the correct risk stratification should be ( ) if he/she has three prognostic risk factors: smoking, obesity, and dyslipidemia

(A) Low risk

(B) Moderate risk

(C) High risk

(D) Very high risk

(E) Cannot be determined

5. For the blood pressure measuring procedure, which of the following descriptions is incorrect?(*You can choose more than one answer*.)

(A) A mercury sphygmomanometer or an upper arm electronic blood pressure monitor that complies with international standards should be used.

(B) The subject rests for at least 2 minutes before the measurement, and the subject is in a sitting position during measurement while keeping quiet and relaxed.

(C) The cuff wraps the upper arm evenly, the lower edge is 2 to 3 cm above the cubital fossa and at the same level as the heart.

(D) During auscultation, systolic blood pressure is when the first korotkoff sound is heard and the diastolic blood pressure is when the fourth korotkoff sound is heard.

(E) The time interval between two blood pressure measurements is 1 to 2 minutes.

6. The main goal of hypertension treatment is to reduce the blood pressure to certain standards. Which of the following blood pressure control standards is correct?

(A) The blood pressure of the average patient with hypertension should be reduced to below 130/80mmHg.

(B) The blood pressure of elderly patients (age ≥65 years) with hypertension should be reduced to below 150/90mmHg.

(C) The blood pressure of patients with diabetes, cerebrovascular disease, and chronic kidney disease should be reduced to below 140/90mmHg.

(D) Blood pressure of patients with all grades of hypertension should reach the control standard within 1 to 2 weeks, and long-term blood pressure control should be achieved.

(E) Patients with coronary heart disease or elderly patients (age >65 years) require attention when the diastolic blood pressure is lower than 50mmHg

7. Non-drug treatment for hypertensive patient includes().(*You can choose more than one answer*.)

(A) Weight loss; body mass index (BMI) should be controlled to <24 kg/m^2^

(B) Regular exercise, which is generally low or moderate intensity exercise, 3 to 5 times a week, 30 minutes per time.

(C) Reduce sodium intake; the daily sodium intake should not exceed 6 grams.

(D) Smoking cessation and limiting alcohol consumption; daily alcohol consumption should not exceed the amount equal to 50 grams of ethanol.

(E) Reduce fat intake; the energy provided by dietary fat should be less than 40% of the total calories consumed.

8. Among the following principles of hypertension drug treatment, which is correct?

(A) Start the treatment from a smaller effective dose, gradually increase the dose or add combined drugs; the goal is to lower the blood pressure to the standard within 1 to 2 weeks.

(B) Intermediate- or short-acting drugs are recommended to lower the blood pressure as soon as possible to prevent target organ damage.

(C) Try to apply only one drug to avoid the adverse effects of antihypertensive drugs.

(D) Following the principle of individualized treatment, choose appropriate antihypertensive drugs according to the specific circumstances of each patient.

(E) Diuretics are preferred in elderly hypertensive patients

9. Contraindications of angiotensin II receptor blocker (ARB) include:()(*You can choose more than one answer*.)

(A) Congestive heart failure

(B) Tachyarrhythmias

(C) Pregnancy

(D) Hyperkalemia

(E) Bilateral renal artery stenosis

10. Indications of the dihydropyridine calcium antagonists for the treatment of hypertension are().(*You can choose more than one answer*.)

(A) Elderly patients (age >65 years) with hypertension

(B) Supraventricular tachycardia

(C) Isolated systolic hypertension

(D) Cardiac angina

(E) Left ventricular dysfunction

11. Which of the following is the taboo against the use of diuretics (anti-aldosterone)?

(A) Elderly patients (age >65 years) with hypertension

(B) Congestive heart failure

(C) Post-myocardial infarction

(D) Diabetic nephropathy

(E) Renal failure

12. Which of the following is correct regarding β-blockers?

(A) There are three types of β-blockers: selective (β1), non-selective (β1 and β2) and those with combined α receptor blockage.

(B) They have better efficacy for elderly patients (age >65 years) with hypertension.

(C) They can only lower the blood pressure in the resting state.

(D) Non-selective β-blockers are preferred in the treatment of hypertension in clinical practice.

(E) The main adverse effect is orthostatic hypotension.

13. Which of the following combination regimens of antihypertensive drugs is inappropriate?

(A) Calcium antagonists and angiotensin-converting enzyme inhibitors (ACEI) or ARB

(B) Small dose of diuretic and ACEI or ARB

(C) ACEI and ARB

(D) Dihydropyridine calcium antagonists and small doses of beta blockers

(E) Calcium antagonists and small doses of diuretics

14. Which of the following hypertension-related treatments is correct?

(A) With continuously elevated blood pressure and serum total cholesterol (TC) levels and TC ≥ 5.2 mmol / L, lipid-lowering therapy using statins should be considered, and the target TC is <4.1mmol / L.

(B) For patients with hypertension and coronary heart disease, diabetes, ischemic stroke, peripheral vascular disease and serum total cholesterol (TC) ≥ 4.1mmol / L, blood lipid-lowering therapy using statins should be considered, and the target TC is <3.1mmol / L.

(C) For patients with hypertension and ischemic heart and cerebrovascular diseases (coronary heart disease, ischemic stroke), or diabetes, daily aspirin at a dose of 75 ~ 100mg is recommended.

(D) For patients with hypertension and type 2 diabetes, the target blood pressure is <140/90mmHg.

(E) For patients with hypertension and type 2 diabetes, the target blood glucose is: fasting blood glucose ≤ 10 mmol / L, glycosylated hemoglobin (HbA 1c) ≤ 6.0%

15. Populations susceptible to hypertension are()?(*You can choose more than one answer.*)

(A) High blood pressure values (systolic blood pressure 130 ~ 139mmHg and / or diastolic blood pressure 85 ~ 89mmHg)

(B) Overweight (BMI 24 ~ 27.9kg/m2) or obese (BMI ≥ 28 kg/m2), and / or abdominal obesity: waist circumference, men ≥ 90cm (2.7 feet), women ≥ 85cm (2.5 feet)

(C) Family history of hypertension(first and second degree relatives)

(D) Long-term excessive alcohol consumption(daily consumption ≥ 200ml / 40 grams)

(E) Age ≥ 50 years

16. Which is correct regarding the management grade of hypertensive patients by general practitioners in the community?

(A) Low-risk patients receive first-grade management and drug therapy is started immediately. They are followed once every 6 months after the blood pressure reaches the standard and is stable.

(B) Patients with moderate risk receive second-grade management and drug therapy is started immediately. They are followed once per month and the blood pressure is monitored if it did not reach the standard or is unstable.

(A) (C) Patients with high risk receive third-grade management and drug therapy is started immediately. Renal function, blood glucose, and lipid profile are measured and urinalysis and electrocardiogram are done once every two years.

(C) Patients with hypertension and comorbidities such as heart, brain and kidney diseases and diabetes are classified as high risk, therefore the management grade will remain unchanged long term.

(D) For patients with target organ damage who are managed by grade, the management grade can be adjusted based on the actual situation after 1 year of management.

17. The referral criteria for the newly diagnosed hypertensive patients in the community include:()(*You can choose more than one answer*.)

(A) Young hypertensive patients

(B) Elderly (age >65 years) hypertensive patients

(C) Pregnant and breast-feeding women

(D) Patients with severe combined clinical conditions or target organ damage

(E) Patients with suspected secondary or white coat hypertension

18. Which one is correct among the following referral criteria for patients with hypertension who are followed up at community health stations?

(A) The treatment regimen was followed for 2-3 weeks, but the blood pressure did not reach the goal.

(B) Patients with stable blood pressure control who experience an increase in blood pressure

(C) Patients who experience adverse reactions after administration of antihypertensive drugs

(D) Patient requires referral to higher level hospitals

(E) Patients with great blood pressure fluctuations or combined multiple risk factors whose clinical treatment is difficult

19. Which one is correct regarding ambulatory blood pressure monitoring?

(A) Ambulatory blood pressure monitoring can be used for diagnosis of white-coat hypertension, masked hypertension, refractory hypertension, paroxysmal hypertension or hypotension.

(B) Normal ambulatory blood pressure values in China are: 24-h average <130/80mmHg, daytime average <140/90mmHg, night time average <120/70mmHg.

(C) Under normal circumstances, the average nighttime blood pressure is 15% to 20% less than the average daytime blood pressure.

(D) Ambulatory blood pressure monitoring is mainly used for the assessment of prognosis, the efficacy of new drugs or treatment regimens, and it gradually replaces the blood pressure measurement at clinics.

(E) The time interval for blood pressure measurement is generally set at once every 60 minutes during the day and once every 120 minutes at night.

## Competing interests

The authors declare that they have no competing interests.

## Authors’ contributions

We declare that all the listed authors have participated actively in the study and all meet the requirements of the authorship. Dr. (QC, SZ) designed the study and wrote the protocol, Dr. (QC, XZ, JG) performed research/study, Dr. (TW, YZ) managed the literature searches and analyses, Dr. (QC, JG) undertook the statistical analysis, Dr. (QC, XZ) wrote the first draft of the manuscript. All authors read and approved the final manuscript.

## Authors’ information

Qian Chen and Xiangjie Zhang are co-first authors in this study.

## Pre-publication history

The pre-publication history for this paper can be accessed here:

http://www.biomedcentral.com/1471-2296/14/16/prepub

## Supplementary Material

Additional file 1 **Table S1. **Responses for knowledge of hypertension.
Click here for file
