# Supplementary material for: General practitioners’ hypertension knowledge and training needs: a survey in Xuhui district, Shanghai
Source: BMC Fam Pract. 2013 Jan 28;14:16. doi: 10.1186/1471-2296-14-16 (PMC3565892; doi:10.1186/1471-2296-14-16)
Supplement: Additional file 1 — Table S1. Responses for knowledge of hypertension. [file 1471-2296-14-16-S1.doc]

**Appendix B**

**Supplementary Table 1**

Table S1. Responses for knowledge of hypertension

| **Domains and questions** | n (%)^a^ |
| --- | --- |
| ***Epidemiology*** |  |
| 1. The epidemiological data show that the current prevalence of hypertension in China is? |  |
| (A) 50 million | 13 (8.8) |
| (B) 100 million | 11 (7.5) |
| (C) 160 million | 40 (27.2) |
| (D) 200 million* | 65 (44.2) |
| (E) 250 million | 10 (6.8) |
| ***Diagnosis*** |  |
| 2. Which of the following is correct about the diagnosis of hypertension? |  |
| (A) For patients without the use of any antihypertensive drugs, hypertension can be diagnosed if two measurements on different days show systolic blood pressure> 140mmHg and / or diastolic blood pressure> 90mmHg. | 65 (44.2) |
| (B) Systolic blood pressure> 140mmHg and diastolic blood pressure ≤ 90mmHg is isolated systolic hypertension. | 0 (0.0) |
| (C) Systolic blood pressure <140mmHg and diastolic blood pressure> 90mmHg is isolated diastolic hypertension. | 0 (0.0) |
| (D) When grading hypertension, if the patient’s systolic and diastolic blood pressure belongs to different grades, a lower grade should be selected. | 1 (0.7) |
| (E) At the same time as diagnosing hypertension, the risk should be stratified according to patient’s blood pressure levels, existing risk factors, target organ damage, and combined clinical disorders.* | 67 (45.6) |
| 3. Which of the following is correct about detecting patients with hypertension in communities? *(You can choose more than one answer.)* |  |
| (A) At all levels of medical institutions, blood pressure should be measured at the first visit of patients over age 35 years.* | 144 (98) |
| (B) For populations susceptible to hypertension (BP 130-139/85-89mmHg, obesity, etc.), it is recommended that blood pressure should be measured every six months. * | 122 (83) |
| (C) A variety of public places should be used to measure blood pressure, including elderly activity stations, clinics at institutions and blood pressure measuring stations. * | 118 (80.3) |
| (D) Make plans to measure the blood pressure of all adults in the community, and blood pressure of adults with normal blood pressure should be measured at least once every 2 years. * | 96 (65.3) |
| (E) Place semi-automatic or automatic electronic blood pressure monitors at a variety of public places to facilitate the public’s self-measurement of blood pressure. * | 88 (59.9) |
| 4. For a patient with hypertension whose blood pressure is 165/95mmHg, the correct risk stratification should be ( ) if he/she has three prognostic risk factors: smoking, obesity, and dyslipidemia |  |
| (A) Low risk | 0 (0.0) |
| (B) Moderate risk | 6 (4.08) |
| (C) High risk* | 114 (77.6) |
| (D) Very high risk | 26 (17.7) |
| (E) Cannot be determined | 1 (0.7) |
| 5. For the blood pressure measuring procedure, which of the following descriptions is incorrect?（*You can choose more than one answer*.） |  |
| (A) A mercury sphygmomanometer or an upper arm electronic blood pressure monitor that complies with international standards should be used. | 25 (17.0) |
| (B) The subject rests for at least 2 minutes before the measurement, and the subject is in a sitting position during measurement while keeping quiet and relaxed.* | 89 (60.5) |
| (C) The cuff wraps the upper arm evenly, the lower edge is 2 to 3 cm above the cubital fossa and at the same level as the heart. | 27 (18.4) |
| (D) During auscultation, systolic blood pressure is when the first korotkoff sound is heard and the diastolic blood pressure is when the fourth korotkoff sound is heard.* | 85 (57.8) |
| (E) The time interval between two blood pressure measurements is 1 to 2 minutes. | 67 (45.6) |
| 6. The main goal of hypertension treatment is to reduce the blood pressure to certain standards. Which of the following blood pressure control standards is correct? |  |
| (A) The blood pressure of the average patient with hypertension should be reduced to below 130/80mmHg. | 21 (14.3) |
| (B) The blood pressure of elderly patients (age ≥65 years) with hypertension should be reduced to below 150/90mmHg.* | 73 (49.7) |
| (C) The blood pressure of patients with diabetes, cerebrovascular disease, and chronic kidney disease should be reduced to below 140/90mmHg. | 2 (1.4) |
| (D) Blood pressure of patients with all grades of hypertension should reach the control standard within 1 to 2 weeks, and long-term blood pressure control should be achieved. | 6 (4.1) |
| (E) Patients with coronary heart disease or elderly patients (age >65 years) require attention when the diastolic blood pressure is lower than 50mmHg | 17 (11.6) |
| 7. Non-drug treatment for hypertensive patient includes（ ）.（*You can choose more than one answer*.） |  |
| (A) Weight loss; body mass index (BMI) should be controlled to <24 kg/m^2^* | 141 (96) |
| (B) Regular exercise, which is generally low or moderate intensity exercise, 3 to 5 times a week, 30 minutes per time. * | 139 (94.6) |
| (C) Reduce sodium intake; the daily sodium intake should not exceed 6 grams. * | 146 (99.3) |
| (D) Smoking cessation and limiting alcohol consumption; daily alcohol consumption should not exceed the amount equal to 50 grams of ethanol. * | 125 (85) |
| (E) Reduce fat intake; the energy provided by dietary fat should be less than 40% of the total calories consumed. | 100 (68) |
| ***Treatment*** |  |
| 8. Among the following principles of hypertension drug treatment, which is correct? |  |
| (A) Start the treatment from a smaller effective dose, gradually increase the dose or add combined drugs; the goal is to lower the blood pressure to the standard within 1 to 2 weeks. | 8 (5.4) |
| (B) Intermediate- or short-acting drugs are recommended to lower the blood pressure as soon as possible to prevent target organ damage. | 0 (0.0) |
| (C) Try to apply only one drug to avoid the adverse effects of antihypertensive drugs. | 1 (0.7) |
| (D) Following the principle of individualized treatment, choose appropriate antihypertensive drugs according to the specific circumstances of each patient.* | 104 (70.8) |
| (E) Diuretics are preferred in elderly hypertensive patients | 3 (2) |
| 9. Contraindications of angiotensin II receptor blocker (ARB) include:（ ）（*You can choose more than one answer*.） |  |
| (A) Congestive heart failure | 8 (5.4) |
| (B) Tachyarrhythmias | 139 (94.6) |
| (C) Pregnancy* | 146 (99.3) |
| (D) Hyperkalemia* | 125 (85) |
| (E) Bilateral renal artery stenosis* | 100 (68) |
| 10. Indications of the dihydropyridine calcium antagonists for the treatment of hypertension are（ ）.（*You can choose more than one answer*.） |  |
| (A) Elderly patients (age >65 years) with hypertension* | 127 (86.4) |
| (B) Supraventricular tachycardia | 23 (15.7) |
| (C) Isolated systolic hypertension* | 105 (71.4) |
| (D) Cardiac angina* | 116 (78.9) |
| (E) Left ventricular dysfunction | 41 (27.9) |
| 11. Which of the following is the taboo against the use of diuretics (anti-aldosterone)? |  |
| (A) Elderly patients (age >65 years) with hypertension | 10 (6.8) |
| (B) Congestive heart failure | 1 (0.7) |
| (C) Post-myocardial infarction | 6 (4.1) |
| (D) Diabetic nephropathy | 34 (23.1) |
| (E) Renal failure* | 71 (48.3) |
| 12. Which of the following is correct regarding β-blockers? |  |
| (A) There are three types of β-blockers: selective (β1), non-selective (β1 and β2) and those with combined α receptor blockage.* | 98 (66.7) |
| (B) They have better efficacy for elderly patients (age >65 years) with hypertension. | 9 (6.1) |
| (C) They can only lower the blood pressure in the resting state. | 7 (4.8) |
| (D) Non-selective β-blockers are preferred in the treatment of hypertension in clinical practice. | 12 (8.2) |
| (E) The main adverse effect is orthostatic hypotension. | 9 (6.1) |
| 13. Which of the following combination regimens of antihypertensive drugs is inappropriate? |  |
| (A) Calcium antagonists and angiotensin-converting enzyme inhibitors (ACEI) or ARB | 2 (1.4) |
| (B) Small dose of diuretic and ACEI or ARB | 9 (6.1) |
| (C) ACEI and ARB* | 109 (74.2) |
| (D) Dihydropyridine calcium antagonists and small doses of beta blockers | 20 (13.6) |
| (E) Calcium antagonists and small doses of diuretics | 0 (0.0) |
| 14. Which of the following hypertension-related treatments is correct? |  |
| (A) With continuously elevated blood pressure and serum total cholesterol (TC) levels and TC ≥ 5.2 mmol / L, lipid-lowering therapy using statins should be considered, and the target TC is <4.1mmol / L. | 17 (11.6) |
| (B) For patients with hypertension and coronary heart disease, diabetes, ischemic stroke, peripheral vascular disease and serum total cholesterol (TC) ≥ 4.1mmol / L, blood lipid-lowering therapy using statins should be considered, and the target TC is <3.1mmol / L. | 25 (17) |
| (C) For patients with hypertension and ischemic heart and cerebrovascular diseases (coronary heart disease, ischemic stroke), or diabetes, daily aspirin at a dose of 75 ~ 100mg is recommended.* | 80 (54.4) |
| (D) For patients with hypertension and type 2 diabetes, the target blood pressure is <140/90mmHg. | 7 (4.8) |
| (E) For patients with hypertension and type 2 diabetes, the target blood glucose is: fasting blood glucose ≤ 10 mmol / L, glycosylated hemoglobin (HbA 1c) ≤ 6.0% | 0 (0.0) |
| 15. Populations susceptible to hypertension are（ ）?（*You can choose more than one answer.*） |  |
| (A) High blood pressure values ​​(systolic blood pressure 130 ~ 139mmHg and / or diastolic blood pressure 85 ~ 89mmHg）* | 123 (83.7) |
| (B) Overweight (BMI 24 ~ 27.9kg/m2) or obese (BMI ≥ 28 kg/m2), and / or abdominal obesity: waist circumference, men ≥ 90cm (2.7 feet), women ≥ 85cm (2.5 feet) * | 142 (96.6) |
| (C) Family history of hypertension（first and second degree relatives）* | 142 (96.6) |
| (D) Long-term excessive alcohol consumption（daily consumption ≥ 200ml / 40 grams） | 103 (70.1) |
| (E) Age ≥ 50 years | 61 (41.5) |
| 16. Which is correct regarding the management grade of hypertensive patients by general practitioners in the community? |  |
| (A) Low-risk patients receive first-grade management and drug therapy is started immediately. They are followed once every 6 months after the blood pressure reaches the standard and is stable. | 11 (7.5) |
| (B) Patients with moderate risk receive second-grade management and drug therapy is started immediately. They are followed once per month and the blood pressure is monitored if it did not reach the standard or is unstable. | 17 (11.6) |
| (C) Patients with high risk receive third-grade management and drug therapy is started immediately. Renal function, blood glucose, and lipid profile are measured and urinalysis and electrocardiogram are done once every two years. | 14 (9.5) |
| (D) Patients with hypertension and comorbidities such as heart, brain and kidney diseases and diabetes are classified as high risk, therefore the management grade will remain unchanged long term.* | 32 (21.8) |
| (E) For patients with target organ damage who are managed by grade, the management grade can be adjusted based on the actual situation after 1 year of management. | 54 (36.7) |
| ***Referral*** |  |
| 17. The referral criteria for the newly diagnosed hypertensive patients in the community include:（ ）（*You can choose more than one answer*.） |  |
| (A) Young hypertensive patients | 38 (25.8) |
| (B) Elderly (age >65 years) hypertensive patients | 9 (6.1) |
| (C) Pregnant and breast-feeding women* | 122 (83) |
| (D) Patients with severe combined clinical conditions or target organ damage* | 138 (93.9) |
| (E) Patients with suspected secondary or white coat hypertension* | 80 (54.4) |
| 18. Which one is correct among the following referral criteria for patients with hypertension who are followed up at community health stations? |  |
| (A) The treatment regimen was followed for 2-3 weeks, but the blood pressure did not reach the goal. | 2 (1.4) |
| (B) Patients with stable blood pressure control who experience an increase in blood pressure | 0 (0.0) |
| (C) Patients who experience adverse reactions after administration of antihypertensive drugs | 0 (0.0) |
| (D) Patient requires referral to higher level hospitals | 0 (0.0) |
| (E) Patients with great blood pressure fluctuations or combined multiple risk factors whose clinical treatment is difficult* | 108 (73.5) |
| ***Community Management*** |  |
| 19. Which one is correct regarding ambulatory blood pressure monitoring? |  |
| (A) Ambulatory blood pressure monitoring can be used for diagnosis of white-coat hypertension, masked hypertension, refractory hypertension, paroxysmal hypertension or hypotension.* | 82 (55.8) |
| (B) Normal ambulatory blood pressure values in China are: 24-h average <130/80mmHg, daytime average <140/90mmHg, night time average <120/70mmHg. | 34 (23.1) |
| (C) Under normal circumstances, the average nighttime blood pressure is 15% to 20% less than the average daytime blood pressure. | 9 (6.1) |
| (D) Ambulatory blood pressure monitoring is mainly used for the assessment of prognosis, the efficacy of new drugs or treatment regimens, and it gradually replaces the blood pressure measurement at clinics. | 4 (2.7) |
| (E) The time interval for blood pressure measurement is generally set at once every 60 minutes during the day and once every 120 minutes at night. | 2 (1.4) |
